# Volumetric Response of Limited Brain Metastatic Disease to Focal Hypofractionated Radiation Therapy

**DOI:** 10.3390/brainsci11111457

**Published:** 2021-11-02

**Authors:** Asanka R. Wijetunga, Dasantha T. Jayamanne, Jessica Adams, Michael F. Back

**Affiliations:** 1 Northern Clinical School, The University of Sydney, St Leonards, NSW 2065, Australia; dasantha.jayamanne@health.nsw.gov.au (D.T.J.); michael.back@health.nsw.gov.au (M.F.B.); 2 Department of Radiation Oncology, Northern Sydney Cancer Centre, St Leonards, NSW 2065, Australia; jessica.adams1@health.nsw.gov.au; 3 Department of Radiation Oncology, Central Coast Cancer Centre, Gosford, NSW 2250, Australia; 4 The Brain Cancer Group, North Shore Private Hospital, St Leonards, NSW 2065, Australia

**Keywords:** brain metastasis, hypofractionated radiotherapy, volume response, radiation necrosis, overall survival

## Abstract

*Background*: This is a retrospective study aimed at assessing the volumetric response, morbidity and failure rates of hypofractionated radiation therapy (HFRT) for definitive focal management of limited brain metastasis. *Methods*: Patients managed with HFRT for unresected limited metastatic (≤10 lesions) brain disease were entered into an ethics-approved database. Included patients had been deemed unsuitable for surgical resection, and lesions managed with prior radiation therapy were excluded. HFRT was delivered using IMRT or VMAT with 25 Gy or 30 Gy in five fractions. Individual lesions had volumetric assessment performed at three timepoints. The primary endpoint was the change of volume from baseline (GTV0) to one month post-HFRT (GTV1) and to seven months post-HFRT (GTV7). Secondary endpoints were local failure, survival and rates of radiation necrosis. *Results*: One hundred and twenty-four patients with 233 lesions were managed with HFRT. Median follow-up was 23.5 months with 32 (25.8%) patients alive at censure. Median overall survival was 7.3 months with 36.3% survival at 12 months. Superior survival was predicted by smaller GTV0 (*p* = 0.003) and increased percentage of volumetric response (*p* < 0.001). Systemic therapy was delivered to 81.5% of patients. At one month post-HFRT, 206 metastases (88.4%) were available for assessment and at seven months post-HFRT, 118 metastases (50.6%) were available. Median metastasis volume at GTV0 was 1.6 cm^3^ (range: 0.1–19.1). At GTV1 and GTV7, this reduced to 0.7 cm^3^ (*p* < 0.001) and 0.3 cm^3^ (*p* < 0.001), respectively, correlating to percentage reductions of 54.9% and 83.3%. No significant predictors of volumetric response following HFRT were identified. Local failure was identified in 4.3% of lesions and radiation necrosis in 3.9%. *Conclusion*: HFRT is an effective therapy for limited metastatic disease in the brain to maximise initial volumetric response whilst minimising toxicity.

## 1. Introduction

Brain metastasis (BM) is the most common intracranial complication of systemic cancer [1,2]. Incidence of BM is increasing due to improved magnetic resonance imaging (MRI) surveillance and improved systemic therapy, increasing the demand for BM management strategies [3,4]. Improved intracranial control directly benefits quality of life, neurocognitive function and possibly overall survival (OS) [1]. Earlier detection also means that management options need to balance durable intracranial control and treatment-related morbidity. As a result, focal therapy of metastatic brain disease using stereotactic radiosurgery (SRS), with or without surgery, and subsequent imaging surveillance is now established in clinical practice [5,6,7].

Whilst SRS is historically the preferred radiation modality, there are concerns over potential morbidity. Stereotactic radiosurgery has been associated with inflammatory complications such as early pseudoprogression or late radiation necrosis which are more pronounced with larger-volume lesions or when co-prescribed with systemic therapies such as immunotherapy [8,9]. Hypofractionated radiotherapy (HFRT), delivered in 3–5 fractions, has the potential, through smaller doses per fraction, to minimize the risk of toxicity compared with single-fraction SRS whilst permitting the delivery of equivalent biologic doses [10]. A study of 289 patients comparing SRS and HFRT to unresected BMs found significantly improved local control (89% vs. 80%, *p* = 0.004) and reduced radiation necrosis (9% vs. 18%, *p* = 0.01) in the HFRT group [11].

This retrospective study aims to report the volumetric response and subsequent outcomes of unresected limited BM following treatment with HFRT to assess the efficacy of this emerging radiation delivery technique and guide clinical decision making.

## 2. Materials and Methods

### 2.1. Patient Selection

Patients diagnosed with unresected limited brain metastatic disease referred to radiation oncology at two university teaching hospitals between January 2014 and July 2020 were entered into an ethics-approved prospective register (ref. LNR/15/HAWKE/355). Limited metastatic brain disease was defined as 10 or fewer BMs [12]. Patients with lesions deemed unsuitable for neurosurgical resection by multidisciplinary team (MDT) consensus due to BM number, size, location, or patient performance status were included in the cohort. Patients with leptomeningeal disease and lesions treated with prior radiation therapy, including WBRT, were excluded from analysis. All lesions in each patient received a uniform modality and dose of radiotherapy, hence small lesions <10 mm were treated with HFRT rather than SRS if there was a concurrent larger lesion being treated.

### 2.2. Baseline Characteristics

Baseline information collected included patient demographics; primary tumour histology, presence of extracranial disease and prior systemic therapy; the date of initial BM diagnosis and ECOG performance status at BM intervention.

Concurrent systemic therapy was defined as chemotherapy, immunotherapy, targeted therapy or a combination of these started within one month of commencement of HFRT.

### 2.3. HFRT Protocol

All patients were treated by one of two experienced specialist neuro-radiation oncologists (DTJ and MFB). Patients were immobilised in either an Orfit mask or a frameless Brainlab cranial mask fixation system, and computed tomography (CT) images were acquired with slice thickness of 1 mm. The diagnostic gadolinium-enhanced MRI was fused within the treatment planning system and rigid registration undertaken. Image fusion accuracy was assessed by comparing both normal tissue structures and metastases on CT with the MRI-defined structures.

HFRT was delivered using an intensity-modulated radiation therapy (IMRT) or a volumetric-modulated arc therapy (VMAT) technique on Varian Trilogy or Varian TrueBeam STX 6 MV linear accelerators. The standard dose fractionation prescription was 30 Gy in 5 fractions delivered in three fractions per week. An integrated boost approach to 25 Gy was utilised for margin expansion to optimise the dose wash around the high-dose region, with 30 Gy dosed to the 100% isodose and delivered to the gadolinium-enhanced lesion on the MRI (GTV), expanded by 2 mm (PTV30); and 25 Gy was delivered to that volume with an additional 3 mm margin (PTV25). Alternative regimens of 25 Gy in 5 fractions or 21 Gy in 3 fractions were utilised in target-overlapped dose-limiting structures or poor patient performance status. Dosage to the optic chiasm and brainstem were limited to 20 Gy and 25 Gy, respectively. Image guidance for each fraction involved cone beam CT.

### 2.4. Volumetric Assessment

Individual BM volumetric assessment (in cm^3^) was recorded at three timepoints: baseline pre-HFRT (GTV0); at one month following HFRT (GTV1); and at six-to-eight months post-HFRT (GTV7). This involved delineating the gadolinium-enhanced lesion within the radiation oncology planning system or in the diagnostic imaging PACS system. These time frames were selected as fixed points for post-treatment MRI surveillance, with patients receiving follow-up scans every three months after their initial one-month review. In the event of clinical deterioration, interval imaging was recommended to exclude the presence of progressive disease or treatment-related morbidity.

### 2.5. Study Endpoints

The primary endpoint was the median change in individual BM volume from GTV0 at one and seven months post-HFRT (GTV1 and GTV7). Volume reduction was calculated as a percentage of GTV0, with a negative value indicative of an increase in size. Secondary endpoints assessed were subsequent local failure, OS, cause of death and rate of radiation necrosis.

Any enhancement was assessed by consensus of the neuro-oncology MDT at the time of occurrence and classed into pseudoprogression, radiation necrosis or local failure after referral for MRI and positron emission tomography. Local failure was defined as a progressive increase in BM volume on two sequential MRI scans without a reduction in volume in subsequent scans and deemed not to be due to the effects of radiotherapy. If death occurred from an extracranial cause before subsequent MRI could show resolution, the event was recorded as unconfirmed. Local control in other patients was defined as being alive without progression or absence of progression on the last MRI or CT image prior to death. The toxicity outcomes recorded from treatment included either pseudoprogression (acute) or radiation necrosis (late), as above.

### 2.6. Statistical Analysis

Descriptive statistics illustrated the baseline features of the study cohort, tumour characteristics and the type of treatments received by patients. GTV0 was compared on a per-lesion basis to the post-treatment timepoints using a paired-sample *t*-test. Factors impacting change in BM volume at one and seven months post-HFRT were determined through linear regression modelling. Survival curves for study outcomes were generated using Kaplan–Meier analysis of total patient BM bulk data. The log-rank test evaluated univariate, and Cox regression analysis with stepwise variable selection evaluated independent predictors of survival and progression. A *p*-value of <0.05 was considered statistically significant. Statistical analyses were performed using IBM SPSS Version 26.

## 3. Results

### 3.1. Demographic Data

Between January 2014 and July 2020, 124 patients with 233 unresected lesions managed with HFRT were entered into the ethics-approved database. Patient demographic data are detailed in Table 1. The median age was 69.3 years (range 35.1–93.9 years). The lung was the commonest primary tumour site (43.5%). The median time from initial BM diagnosis to HFRT was 1.0 months (range 0.0–54.2 months). The variability may be explained by 10 (8.1%) patients being managed medically for over one year before being considered for radiotherapy.

### 3.2. Patient Treatments and Outcomes

The treatment and outcome data of the 124 included patients are presented in Table 2. Concurrent systemic therapy was delivered in 81.5% of patients. Following upfront therapy, patients were most commonly managed with supportive care alone (*n* = 74, 59.7%), with only 22 (17.7%) being managed with systemic therapy. The median percentages of volume reduction per patient at the two follow-up timepoints were 48.5% (range −304.4–100.0%) and 80.6% (range −328.6–100.0%), respectively.

**Figure 1:**
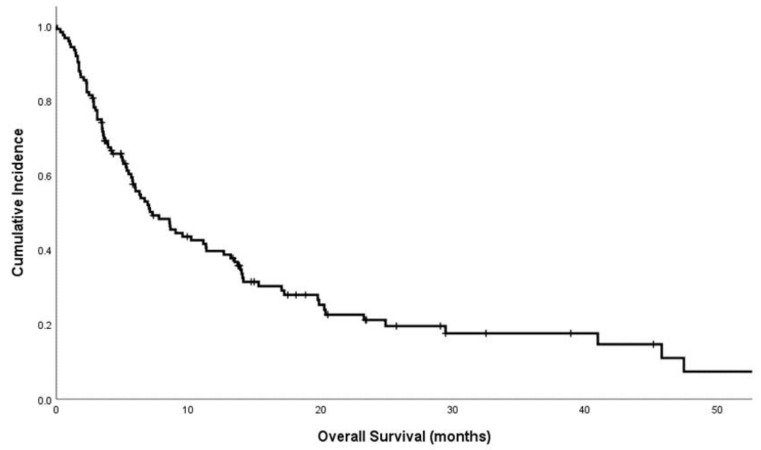
Kaplan–Meier plot of survival of the cohort of 124 patients. Median overall survival is 7.3 months. Two-month, six-month and twelve-month survival is 79.8%, 50.0% and 36.3%, respectively.

The median follow-up time was 23.5 months. Thirty-two (25.8%) patients were alive at the date of data censure. The median OS of the cohort was 7.3 months (Figure 1), with 12-month survival being 36.3%. Of the deceased patients, twenty-one (16.9%) died of purely intracranial disease, and seven (5.6%) died of both intracranial and extracranial disease. The remaining 64 patients (51.6%) died from extracranial disease (44.4%) or other causes (7.3%).

**Figure 2:**
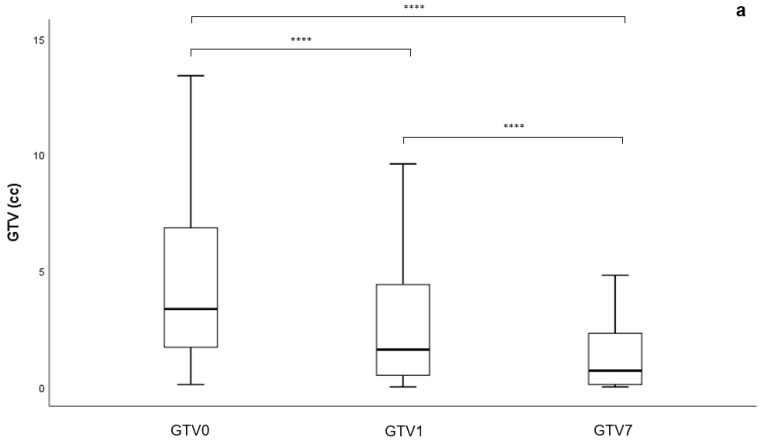

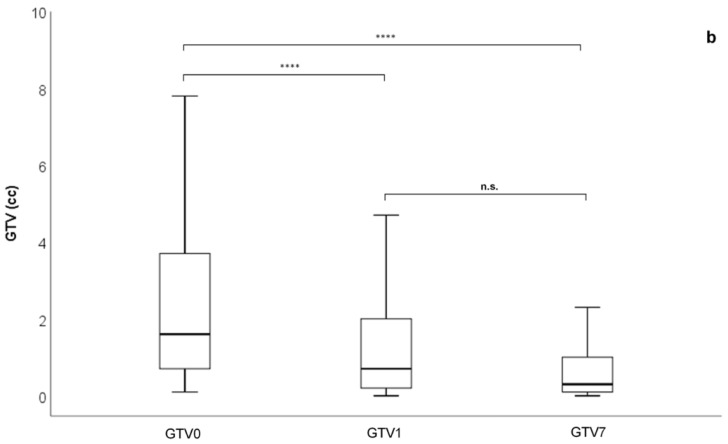
Change in volume (**a**) of total tumour bulk per patient (*N* = 124). Pre-HFRT volume is significantly greater than at one month post-HFRT (*p* < 0.001, ****) and seven months post-HFRT (*p* < 0.001, ****). Volume at one month is significantly greater than at seven months post-HFRT (*p* < 0.001, ****). (**b**) of each metastasis (*N* = 233). Pre-HFRT volume is significantly greater than at one month post-HFRT (*p* < 0.001, ****) and is significantly greater than seven months post-HFRT (*p* < 0.001, ****). Volumes at one month and seven months post-HFRT are not significantly different to each other (*p* = 0.224, n.s.). GTV0 = volume pre-treatment, GTV1 = volume at one month post-HFRT, GTV7 = volume at seven months post-HFRT, HFRT = hypofractionated stereotactic radiotherapy.

The median total BM volume per patient was 3.4 cm^3^ (range 0.1–48.2 cm^3^) pre-HFRT, which fell significantly to 1.6 cm^3^ after one month (range 0.0–45.8 cm^3^, 48.5% reduction, *p* < 0.001) and to 0.7 cm^3^ (range 0.0–24.6 cm^3^, 80.6% reduction, *p* < 0.001) after seven months (Figure 2a).

Local failure was confirmed in only eight patients (6.5%); however, in sixteen (12.9%) patients, it was impossible to determine whether local failure had occurred due to rapid decline from advanced extracranial disease without any further brain imaging. Seven (5.6%) patients experienced a radiation necrosis event (Table 2).

### 3.3. Individual Lesion Treatments and Outcomes

The characteristics of the 233 lesions managed with HFRT are detailed in Table 3. The most common neuroanatomical sites were the frontal lobe (*n* = 60, 25.8%), the cerebellum (*n* = 48, 20.6%) and the parietal lobe (*n* = 42, 18.0%). The 30-Gy HFRT prescription was used for 88.8% of the lesions. The median biologically effective dose for an α/β of 12 (BED12) was 45 Gy.

At one-month post-HFRT, 206 metastases (88.4%) were available for assessment, and at seven months post HFRT, this had reduced to 118 metastases (50.6%).

The median individual BM volume pre-HFRT (GTV0) of the cohort was 1.6 cm^3^ (range: 0.1–19.1 cm^3^). The median GTV1 and GTV7 were 0.7 cm^3^ (range: 0.0–35.1 cm^3^, 54.9% reduction) and 0.3 cm^3^ (range: 0.0–23.8 cm^3^, 83.3% reduction), respectively. The median volumes at both GTV1 and GTV7 timepoints were significantly smaller than GTV0 median volume (*p* < 0.001 and *p* < 0.001, respectively) (Figure 2b).

Thirty-four (14.6%) metastases had a volume increase >0.1 cm^3^ from baseline at one month post-HFRT; subsequently, ten of these (4.3%) were classified as events of pseudoprogression, and 15 (6.4%) died and could not be assessed further. Ten (4.3%) lesions which did not experience initial volume expansion were found to progress between the one- and seven-month scans. Failure was confirmed in a total of ten (4.3%) lesions and radiation necrosis in nine (3.9%), resulting in a local control rate of 95.7%. Among the lesions stated to have achieved local control were 24 (10.3%) metastases defined as unconfirmed.

### 3.4. Factors Associated with Volumetric Endpoints

Univariate linear regression of various factors, including age at diagnosis, GTV0, primary tumour histology, radiation dose and concurrent systemic therapy, showed no significant predictors of the degree of volumetric response following HFRT on an individual metastasis or per-patient basis (Table A1). Specifically, the total and individual GTV0 were not significantly associated with volumetric response at one month (*p* = 0.948, *p* = 0.821, respectively).

Younger age at diagnosis (HR = 0.93, *p* = 0.006) was associated with lesion local failure on individual logistic regression on a per-lesion basis (Table A2), but no other factors were correlated with local failure. The median GTV0 of lesions which locally failed was 4.6 cm^3^ (range 0.9–5.4 cm^3^), not significantly different to the remainder of the cohort (*p* = 0.401). There were no factors identified to be associated with radiation necrosis (Table A2). Specifically, the median GTV0 of metastases which developed radiation necrosis was 2.1 cm^3^ (range 0.7–6.0 cm^3^), not significantly different to the remainder of the cohort (*p* = 0.249).

### 3.5. Factors Associated with Overall Survival

The factors associated with OS are detailed in Table 4. On multivariate Cox regression analysis, factors independently associated with improved OS were smaller initial tumour volume (HR = 2.59, *p* = 0.003); volume reduction of >50% at one month post-HFRT (HR = 3.33, *p* = 0.005); volume reduction >80% at seven months post-HFRT (HR = 0.32, *p* < 0.001) and the use of a combination of more than one modality of systemic therapy concurrently with HFRT (HR = 0.43, *p* = 0.035).

## 4. Discussion

This study demonstrates that the use of HFRT in the management of unresected, limited metastatic disease of the brain confers an early and durable volume reduction without significant risk of adverse events such as radiation necrosis. As there are minimal data to demonstrate the actuarial volume response following HFRT in unresected BMs, this study assists in decision making when considering SRS or HFRT for a larger-volume lesion.

The novelty of this study lies in its presentation of a large cohort of patients with unresected BMs treated with upfront HFRT by two experienced neuro-radiation oncologists working together across two linked cancer centres during a short temporal window from 2014–2020. Furthermore, no size or histopathologic exclusion criteria were applied, improving the generalisability of these findings.

Among the 233 metastases included, the median pre-HFRT volume was 1.6 cm^3^ (range 0.1–19.1 cm^3^), which significantly fell to 0.7 cm^3^ (48.5% reduction from baseline) one month post-HFRT and fell further in another six months to 0.3 cm^3^ (80.6% reduction from baseline). On linear regression, no patient, treatment or tumour factors were correlated with an inferior volume response, demonstrating that HFRT is equally effective at achieving a volumetric response for most BMs irrespective of their initial size, site or histology. This differs from published SRS data, which describe differing outcomes depending on the histopathological primary [13,14].

The volumetric response greater than 80% to HFRT in this study is at least equivalent to published volumetric responses with SRS. Sharpton et al. found a 64% reduction in median volume after three months, and no subsequent volume response was documented [14]. An analysis of 91 lesions treated with SRS alone by Diao et al. showed that the 60% volume reduction from baseline achieved one month post-SRS rebounded by 17% to a 43% reduction from baseline after 6 months [9]. Only in the SRS + immunotherapy arm of Diao et al.’s study was a volume response rate of 80% achieved. In our study, only one-third of the cohort was managed with immunotherapy, and the use of systemic therapy was not associated with improved volumetric response, highlighting the potency of HFRT for BM volume reduction [15].

Rates of transient pseudoprogression appear to be lower in HFRT compared to SRS, with this study finding that only 4.3% of patients experienced initial volume expansion, which resolved by the seven-months-post-HFRT assessment. Pseudoprogression following SRS is postulated to be due to the opening of the blood–brain barrier and the ingress of leukocytes to the treatment site, and it is believed to reflect the high-fraction dose of radiation to the target lesion [8]. Cohorts treated with SRS experienced higher rates of pseudoprogression; the Bergen Criteria definition study of 348 SRS-treated metastases reported pseudoprogression rates of 14% [8]. Furthermore, Sparacia et al. found pseudoprogression in 28% of 54 lesions treated with SRS, which resolved at 12 weeks [16]. Minimising pseudoprogression reduces symptom load and quells the anxiety of patients in whom there may be uncertainty over relapse. Oncologists may also need to delay or alter systemic therapy approaches where pseudoprogression and relapse are indistinguishable, and increase corticosteroid requirements in cases of BM volume expansion, which may impact extracranial disease control [8,17].

Local control rates in this study were greater than 90%, though almost 13% of patients were unable to have response assessment. This is an accepted issue in reporting outcomes in advanced cancer complicated by BM, where there are the competing risks of intracranial and extracranial disease [18]. Two retrospective comparative studies between HFRT and SRS have found that local control rates at 12 months for HFRT were 91% [11] and 70% [19], compared with 77% and 56%, respectively, in SRS, consistent with our study. Minniti et al., who reported 70% local control at 12 months, delivered a dose of HFRT with median BED12 of 47 Gy [11], equivalent to our study.

Despite international guidelines recommending SRS for limited BM in most clinical circumstances [20,21], the biological advantages, efficacy and safety of hypofractionation of radiation doses are established [22,23]. Compared with SRS, fractionation improves radiosensitisation through re-oxygenation of hypoxic malignant cells – augmenting tumour shrinkage through oxidative stress [24]. There is differing radiobiology between normal brain and tumour which can be optimised through HFRT over SRS. Since normal brain is a late-responder to radiation with a low α/β, and BM an early-responder with a high α/β, SRS is more likely than HFRT to damage normal tissue, leading to complications such as radiation necrosis [25]. A HFRT approach with VMAT or IMRT, although treating more surrounding normal brain, may limit the injury to that tissue by allowing maximal recovery time between fractions [26,27]. In our study, radiation necrosis was reported in only 3.9% of metastases during the follow-up period. In Putz et al.’s comparison of HFRT (*n* = 98) and SRS (*n* = 92), a similar necrosis rate of 3.4% was found in the 12 months following HFRT, with a median BED12 of 52.4 Gy administered [28]. Despite a lower median BED12 of 41.0 Gy being used for SRS, the rate of necrosis was significantly higher (14.8%, *p* = 0.045 on multivariate analysis). Rates of necrosis following HRFT are typically <10% [11,19,28,29,30], whilst SRS has been associated with necrosis in over 20% of patients in two studies, each over 250 metastases, by Minniti et al. [11,31]. Furthermore, a meta-analysis of 24 studies comparing SRS vs. HFRT on BMs 2–3 cm in diameter demonstrated significantly less radionecrosis in the HFRT group (23.1% vs. 7.3%, *p* = 0.003), with no difference in LC (77.6% vs. 92.9%, *p* = 0.18), further suggesting that a hypofractionated approach confers a lower risk of radiation toxicity without compromising efficacy [32].

Overall survival in this study was 7.3 months, equivalent to other studies of HFRT in unresected BM: a summary of six cohorts by Murai et al., totalling 363 patients, demonstrated median OS post-HFRT of 3–15 months [33]. The survival of 7.3 months ought to be considered with the fact that initial management for many patients is resection followed by adjuvant cavity radiotherapy [34]. Only those with advanced extracranial disease, multiple lesions or co-morbidity are selected for non-operative management. Despite the range of benefits of HFRT over SRS, neither appear to confer superior survival, with several retrospective comparative studies of 90–190 patients finding no significant difference [19,35,36,37], which may be due to the competing risk of extracranial disease on OS. In our study, HFRT conferred a significant and consistent volumetric response which was independently predictive of superior OS.

Although the data demonstrate a role for HFRT in the management of BM, the logistical impact on departmental workload should be considered, with the benefit of low rates of necrosis, pseudoprogression and local failure being balanced against the increased number of patient attendances required for HFRT over SRS [38] and an increased dose of radiation to normal brain tissue, especially where multiple small-volume BMs are being treated or multiple HFRT courses are required [39]. Improved planning software and delivery techniques may mitigate these risks through improved dosimetry. Additionally, more data are required to prove whether three- and five-fraction regimens provide equivalent outcomes, reducing the logistical impact.

The interpretations from this study are principally limited by the retrospective audit design and the variability of extracranial disease influencing patient selection. There was extensive heterogeneity in the choice of systemic therapy, as patients were being managed by multiple medical oncologists for a range of primary subtypes during an era where immunotherapy and targeted therapies were evolving in clinical practice. Toxicity data are also limited, with an absence of dexamethasone data and diagnosis of late radiation necrosis being dependent upon the timing of the imaging and patient survival. Balancing these features is that all patients were treated uniformly with similar dosing regimens by only two radiation oncologists in units with established follow-up procedures.

## 5. Conclusions

Hypofractionated radiotherapy is an effective method for delivering high-dose radiation to limited metastatic brain disease, maximising initial volumetric response whilst minimising pseudoprogression and radiation necrosis. Volumetric response, an independent predictor of survival, was demonstrated in metastases of all treated primary tumour pathologies, intracranial sites and tumour volumes. The data from this study should provide confidence in decision making for advanced cancer patients with limited brain metastases.

## Figures and Tables

**Table 1:** Demographic data of the 124 cohort patients. ECOG = European Collaborative Oncology Group performance status score.

Demographic Data	*N* = 124
**Age (years)**	
Median (range)	69.3 (35.1–93.9)
**Gender (%)**	
Male	64 (51.6)
Female	60 (48.4)
**Primary tumour location—Number (%)**	
Lung–Non-Small Cell	50 (40.3)
Lung–Small Cell	4 (3.2)
Melanoma	21 (16.9)
Breast	15 (12.1)
Colorectal	8 (6.5)
Oesophageal	2 (1.6)
Renal	8 (6.5)
Others/Unknown Primary	16 (12.9)
**Time from CNS metastasis to radiotherapy (months)**	
Median (range)	1.0 (0.0–54.2)
**Extracranial disease**	
Nil	6 (4.8)
Asymptomatic	51 (41.1)
Symptomatic	67 (54.1)
**ECOG performance status—Number (%)**	
0	9 (7.3)
1	47 (37.9)
2	46 (37.1)
3	21 (16.9)
4	1 (0.8)

**Table 2:** Summary of treatments and volumetric outcomes of the 124 included patients. HFRT = hypofractionated radiotherapy, SRS = stereotactic radiosurgery, VMAT = volumetric modulated arc therapy, WBRT = whole-brain radiotherapy. *A negative value for volume reduction indicates volume expansion.

Treatment and Outcomes	*N* = 124
**Number of metastases per patient at diagnosis**	
Median (range)	1 (1–7)
**HFRT dose—Number (%)**	
30 Gy/5	106 (85.5)
25 Gy/5	9 (7.3)
21 Gy/3	7 (5.6)
Other	2 (1.6)
**Prior management—Number (%)**	
Nil Prior Management	75 (60.5)
Surgery	26 (21.0)
Systemic Therapy	11 (8.9)
Distant Site SRS	3 (2.4)
Combination	9 (7.3)
**Concurrent systemic therapy—Number (%)**	
Nil	23 (18.5)
Chemotherapy Alone	39 (31.5)
Targeted Therapy Alone	12 (9.7)
Immunotherapy Alone	16 (12.9)
Immunotherapy + Chemotherapy	19 (15.3)
Immunotherapy + Targeted Therapy	10 (8.1)
Chemotherapy + Targeted Therapy	5 (4.0)
**Volume at time of diagnosis**	
Median volume (range) (cm^3^)	3.4 (0.1, 48.2)
**Volume one month post-HFRT**	
Number of patients alive—Number (%)	105 (84.6)
Median volume (range) (cm^3^)	1.6 (0.0, 45.8)
Percent reduction—Median (range) (%) *	48.5 (−304.4, 100.0)
**Volume seven months post-HFRT**	
Number of patients alive—Number (%)	67 (54.0)
Median volume (range) (cm^3^)	0.7 (0.0, 24.6)
Percent reduction—Median (range) (%) *	80.6 (−328.6, 100.0)
**Subsequent management—Number (%)**	
Best Supportive Care	74 (59.7)
Surgery	5 (4.0)
SRS/VMAT	13 (10.5)
WBRT	10 (8.1)
Systemic Therapy	22 (17.7)
**Outcome of treatment—Number (%)**	
Radiation necrosis	7 (5.6)
Local failure	8 (6.5)
Unconfirmed	16 (12.9)
**Cause of death—Number (%)**	
Alive	32 (25.8)
Intracranial	21 (16.9)
Extracranial	55 (44.4)
Both	7 (5.6)
Other	9 (7.3)

**Table 3:** Summary of treatments and volumetric outcomes of the 233 individual metastases. HFRT = hypofractionated radiotherapy. * A negative value for volume reduction indicates volume expansion. ** Pseudoprogression.

Individual Lesions Treatments and Outcomes	*N* = 233
**Location—Number (%)**	
Frontal	60 (25.8)
Parietal	42 (18.0)
Temporal	39 (16.7)
Occipital	19 (8.2)
Brainstem	7 (3.0)
Cerebellum	48 (20.6)
Other	18 (7.7)
**Radiation dose—Number (%)**	
30 Gy/5	208 (89.3)
25 Gy/5	10 (4.3)
21 Gy/3	12 (5.2)
Other	3 (1.3)
**At time of diagnosis**	
Number of metastases—Number (%)	233 (100.0)
Median volume (range) (cm^3^)	1.6 (0.1, 19.1)
**One month post-HFRT**	
Number of metastases—Number (%)	206 (88.4)
Median volume (range) (cm^3^)	0.7 (0.0, 35.1)
Percent reduction—Median (range) (%) *	54.9 (−700.0, 100.0)
Volume increase–Number (%)	34 (14.6)
**Seven months post-HFRT**	
Number of metastases—Number (%)	118 (50.6)
Median volume (range) (cm^3^)	0.3 (0.0, 23.8)
Median volume (range) (cm^3^) *	83.3 (−328.6, 100.0)
Volume increase at one month sustained—Number (%)	9 (3.9)
Volume increase at one month transient—Number (%) **	10 (4.3)
Volume increase at one month died—Number (%)	15 (6.4)
**Outcome of treatment—Number (%)**	
Radiation necrosis	9 (3.9)
Local failure	10 (4.3)
Unconfirmed	24 (10.3)

**Table 4:** Factors predictive of overall survival. BM = brain metastasis, GTV0 = volume pre-HFRT, GTV0 = volume at one month post-HFRT, GTV7 = volume at seven months post-HFRT, HFRT = hypofractionated radiotherapy, MV = multivariate analysis with Cox regression, NSCLC = non-small cell lung cancer, OS = overall survival, SCLC = small-cell lung cancer, UV = univariate analysis with Log-rank test.

Factors Associated with Overall Survival			*n* = 124		
	Median OS (Months)	UV HR (95% CI)	UV *p*-Value	MV HR (95% CI)	MV *p*-Value
Sex (M/F)	6.3/11.1	1.44 (0.95–2.18)	0.083	-	-
Age at diagnosis (<75/>75)	8.6/3.6	1.71 (1.09–2.69)	0.017	0.69 (0.33–1.46)	0.336
GTV0 (<3.4 cm^3^/>3.4 cm^3^)	**11.1/6.9**	**1.56 (1.02–2.38)**	**0.038**	**2.59 (1.38–4.86)**	**0.003**
GTV1 (<3.0 cm^3^/>3.0 cm^3^)	13.9/6.9	2.22 (1.44–3.69)	0.002	0.88 (0.34–2.28)	0.787
GTV7 (<0.7 cm^3^/>0.7 cm^3^)	20.4/13.7	2.34 (1.35–4.78)	0.003	1.65 (0.51–5.36)	0.405
GTV1 reduction (<50%/>50%)	**6.9/14.7**	**0.61 (0.38–0.97)**	**0.034**	**3.33 (1.44–7.68)**	**0.005**
GTV7 reduction (<80%/>80%)	**11.3/23.4**	**0.33 (0.17–0.62)**	**<0.001**	**0.32 (0.17–0.61)**	**<0.001**
ECOG (0–1/2–4)	17.0/5.0	2.59 (1.68–4.00)	<0.001	1.27 (0.65–2.49)	0.488
Melanoma primary (Y/N)	5.57.8	1.49 (0.88–2.49)	0.123	-	-
Colorectal primary (Y/N)	5.1/8.6	1.60 (0.82–3.09)	0.161	-	-
NSCLC primary (Y/N)	7.3/7.1	0.87 (0.57–1.32)	0.505	-	-
SCLC primary (Y/N)	6.4/7.8	1.11 (0.35–3.52)	0.863	-	-
Renal primary (Y/N)	8.6/7.0	0.63 (0.23–1.72)	0.362	-	-
Breast primary (Y/N)	13.5/7.0	0.70 (0.35–1.40)	0.316	-	-
Symptomatic extracranial disease (Y/N)	5.3/13.2	2.01 (1.32–3.06)	0.001	1.2 (0.58–2.56)	0.597
Neurosurgery prior to HFRT (Y/N)	13.2/6.7	0.55 (0.32–0.95)	0.030	0.66 (0.31–1.41)	0.287
Systemic therapy prior to HFRT (Y/N)	41.0/7.0	0.43 (0.18–0.99)	0.040	1.01 (0.25–4.10)	0.987
Number of BMs (<1/>1)	8.6/6.0	1.42 (0.87–2.31)	0.158	-	-
Radiation dose 30 Gy/5 (Y/N)	8.6/6.0	0.74 (0.34–1.60)	0.438	-	-
Systemic therapy concurrent with HFRT (Y/N)	8.6/3.5	0.49 (0.30–0.80)	0.003	1.73 (0.62–4.78)	0.293
Concurrent immunotherapy (Y/N)	8.6/6.9	0.74 (0.48–1.16)	0.191	-	-
Concurrent targeted therapy (Y/N)	17.2/6.7	0.50 (0.28–0.87)	0.012	0.65 (0.27–1.58)	0.339
Concurrent chemotherapy (Y/N)	9.0/5.5	0.80 (0.53–1.21)	0.281	-	-
Concurrent combination systemic therapy (Y/N)	**14.1/6.4**	**0.50 (0.30–0.83)**	**0.006**	**0.43 (0.20–0.94)**	**0.035**
Salvage neurosurgery (Y/N)	NR/6.9	0.13 (0.02–0.96)	0.019	0.37 (0.05–2.88)	0.341
Salvage systemic therapy (Y/N)	13.9/6.3	0.60 (0.35–1.05)	0.072	-	-
Salvage radiotherapy (Y/N)	9.0/7,.1	1.04 (0.62–1.76)	0.871	-	-
Local failure (Y/N)	2.5/8.6	2.15 (1.32–3.49)	0.001	1.19 (0.44–3.21)	0.729

## Appendix A

**Table A1:** Factors predicting change in volume at one month post-HFRT on individual linear regression. HFRT = hypofractionated radiotherapy.

	Total Volume		Individual Volume	
Factor	Unstandardised B (95% CI)	*p* Value	Unstandardised B (95% CI)	*p* Value
Age at diagnosis	−0.24 (−1.49–1.01)	0.705	−0.08 (−1.23–1.07)	0.892
Male sex	−0.70 (−31.3–29.90)	0.964	−11.68 (−39.00–15.63)	0.400
GTV0	0.07 (−2.05–2.19)	0.948	0.428 (−3.30–4.16)	0.821
Melanoma primary	−17.43 (−76.79–41.93)	0.561	−23.46 (−74.56–27.64)	0.366
Colorectal primary	−60.89 (−122.46–0.68)	0.053	−28.97 (−82.83–24.90)	0.290
NSCLC primary	−5.31 (−49.84–39.22)	0.813	−10.25 (−48.69–28.18)	0.599
SCLC primary	26.80 (−78.17–131.77)	0.613	44.68 (−33.99–123.35)	0.264
Renal primary	25.95 (−44.60–96.49)	0.467	−6.47 (−67.08–54.15)	0.834
Breast primary	−15.22 (−72.14–41.69)	0.596	−19.47 (−70.00–31.07)	0.448
Radiotherapy dose 30 Gy/5	−4.70 (−60.28–5.87)	0.948	−5.40 (−50.18–39.39)	0.812
Immunotherapy	−11.04 (−44.84–22.76)	0.518	−12.23 (−42.44–17.98)	0.426
Targeted therapy	−17.41 (−57.94–23.13)	0.396	−10.94 (−48.19–26.31)	0.563
Chemotherapy	10.08 (−29.74–49.90)	0.616	1.29 (−33.04–35.53)	0.941
**On Multiple Regression**				
*R* ^2^	0.115		0.039	
F (*p*-value)	0.49 (0.919)		0.61 (0.848)	

**Table A2:** Factors predictive of local failure and radiation necrosis on logistic regression. HFRT = hypofractionated radiotherapy, NSCLC = non-small cell lung cancer.

Predictor	Local Failure		Radiation Necrosis	
	HR (95% CI)	*p*-Value	HR (95% CI)	*p*-Value
Male sex	1.51 (0.41–5.51)	0.530	1.25 (0.33–4.78)	0.744
Age at diagnosis	**0.93 (0.89–0.98)**	**0.006**	1.02 (0.96–1.08)	0.560
Initial volume (cm^3^)	1.08 (0.94–1.25)	0.257	1.07 (0.92–1.24)	0.404
Melanoma primary	1.26 (0.26–6.16)	0.778	1.44 (0.29–7.23)	0.655
NSCLC primary	0.72 (0.18–2.87)	0.645	1.38 (0.36–5.31)	0.634
Breast primary	1.89 (0.38–9.41)	0.435	0.91 (0.11–7.58)	0.912
HFRT dose 30 Gy/5	0.46 (0.09–2.30)	0.344	0.0 (0.0–0.0)	0.997
Systemic therapy with HFRT	1.04 (0.21–5.06)	0.962	0.0 (0.0–0.0)	0.997
